# Nurses’ Perceived Facilitators of Research Utilisation in a Multicultural Setting in Saudi Arabia: Observational Study

**DOI:** 10.3390/nursrep12010017

**Published:** 2022-03-02

**Authors:** Mohammed Saleh Almalki, Amanda Kimpton, Linda Katherine Jones, Cliff Da Costa

**Affiliations:** 1 Nursing Department, Taif University, P.O. Box 11099, Taif 21944, Saudi Arabia; msmalki@tu.edu.sa; 2 School of Health and Biomedical Sciences, Royal Melbourne Institute of Technology University, P.O. Box 71, Bundoora, VIC 3083, Australia; amanda.kimpton@rmit.edu.au (A.K.); cliff.dacosta@rmit.edu.au (C.D.C.)

**Keywords:** research utilisation, evidence-based practice, demographics, nursing, health care

## Abstract

Facilitators of research utilisation are important in the implementation of evidence-based practice. Numerous facilitators for nursing practice have been identified, but knowledge of the impact of demographic characteristics on these enablers of research utilisation is limited. The study’s aim was to determine nurses’ perceptions of the facilitators of research utilisation and assess differences in the facilitator of research utilisation score based on nurses’ demographic characteristics. A total of 2650 registered nurses from five hospitals in Riyadh, Saudi Arabia, were recruited for participation. A facilitator scale and self-designed demographic survey were used for data collection. The number of completed questionnaires was 1824 (69%). The results showed that many of the participants were female, aged between 20 to 40 years, and were expatriates mainly from the Philippines. Most respondents were clinical nurses with 6 to 10 years of experience. Many of the nurses had a bachelor’s degree and a qualification from the Asian region. The mean total facilitator score was 26.1, with strong facilitators of research, including advanced education, providing colleague support, more clinically focused research and employing nurses with research skills. Recommendations for the facilitation of research utilisation include a strengthening of the research curriculum in nursing education programs as well as through continuing professional education.

## 1. Introduction

Evidence-based practice (EBP) is recognised as an important factor in improving the quality of healthcare [1,2]. EBP was first defined by Sackett and colleagues as an integration of clinical expertise, best available evidence and patients’ value, which are translated into an answerable question followed by utilisation of the best available information to answer the question [3]. In other words, EPB forms the umbrella of which research utilisation is a part. For example, a meta-analysis investigated the effectiveness of quality improvement strategies to improve diabetes management; a strategy used was EBP, with improvement in diabetes care and nurses scoring higher than physicians in the administration of these interventions for patients [4]. The practice of EBP is determined by the sources of evidence (research utilisation), experience of the practitioner and desires and expectations of patients [5]. Nurses who practice EBP have been found to be empowered and practice with heightened self-confidence, as they provide care based on evidence rather than routine [6]. Associated with EBP is research utilisation, which, for nursing, is defined as the application of research findings in all aspects of nursing practice [1,2,7]. It is unequivocal that research utilisation and EBP improve nurses’ performance [8,9]. Most nurses appreciate the importance of research utilisation, but also believe they cannot apply it in practice as required [1,2,10].

The need to incorporate EBP into clinical practice is well recognised; however, its application is still impeded by nurses’ perceptions of the barriers to research utilisation [11]. These barriers can include lack of knowledge and skills, limited time, support and resources [11]. Factors found to promote engagement and acceptance of EBP for nurses include organisational support in the provision of sufficient resources and continuing education, especially in a clinical setting [11]. In the United States of America, Cline et al. [8] identified facilitators that may foster the use of research findings amongst nurses, which included support from administration and colleagues and the allocation of enough time for research findings appraisal and implementation. These findings of administrative support and time for nurses to read and analyse research are similar to studies conducted in Northern Ireland [12], Spain [13], China [14] and Australia [11]. In contrast to these findings, Breimaier, Halfens and Lohrmann [15] found that in Austria, the main facilitators for the use of research findings for nurses included increasing knowledge by participation in education courses and allocation of time for nurses to read and access research. Similarly, the education of nurses was found to be one of the main facilitators for studies in Sweden [16], the United Kingdom [9], China [14] and Hong Kong [17].

There is a paucity of research undertaken in the Middle East region to explore facilitators of research utilisation for nurses. Mehrdad, Salsali and Kazemnejad [18] conducted a descriptive quantitative study to identify the facilitators of research utilisation of 410 Iranian nurses and reported the most important facilitators were nursing colleagues’ and nursing faculty support, and time and opportunities to attend nursing conferences. In Saudi Arabia, only one study has been identified that has explored the facilitators of research utilisation in nursing practice [19]. Omer [19] conducted a descriptive study involving 413 nurses from the Saudi National Guard Hospitals located in Riyadh, Jeddah, and Al-Ahsain. Open-ended questions were utilised, requiring nurses to list factors of research utilisation in their practice. The main facilitators identified were growth in administrative support, an increase in the availability of research articles in clinical practice settings and the provision of enough time for nurses to review research relevant to their clinical practice [19]. There were several limitations associated with this study, including a low response rate (34.4%) and hospitals included in the study being limited to National Guard Health Hospitals rather than a variety of hospital types. Furthermore, there was no detailed description of the instrument used to measure research utilisation, and the analysis of the collected data did not include a comparison of facilitators and demographic characteristics [19].

The aim of this study was to determine nurses’ perceptions of the facilitators of research utilisation and to investigate how these facilitators differ according to nurses’ demographics and characteristics.

## 2. Materials and Methods

The study was conducted in Riyadh, Saudi Arabia, at five hospitals, including government and education hospitals. Using a convenience sample, participants were asked to complete a hard copy survey that was available in each ward for distribution. The survey took approximately 15 to 20 min to complete by participants. The inclusion criteria included full-time registered nurses with a minimum of two years’ employment at the hospital. A minimum of two years’ employment ensured that participants were successfully oriented to their hospital’s policies, procedures, protocols and practice guidelines. Non-registered Saudi Arabian nurses, including student nurses and nurses assistants, were excluded from the study. The facilitator scale developed by Hutchinson and Johnston [20] was the principal instrument used in this study with permission. The facilitator scale comprises eight items with a five-point Likert type scale for rating of each item as a facilitator of research utilisation, which included 1 = ‘to no extent’, 2 = ‘to a little extent’, 3 = ‘to a moderate extent’, 4 = ‘to a great extent’, and 5 = ‘no opinion’. The highest possible score for the facilitator scale was 32 if the ‘to a great extent’ category, coded 4, was selected for all eight items. The higher the score for the facilitator scale, the greater these factors are perceived as facilitators of research utilisation. The questionnaire also comprised items that sought demographic data, including age, gender, nationality, years of experience, nursing qualification, place where the highest level of nursing education was achieved and current role. Content validity of the selected instruments was established by a panel of experts, including researchers, academics and professional nurses in Saudi Arabia. The panel assessed whether the items within the instrument were relevant and acceptable to the target population and setting, any need for clarification of the items and the detection of any concerns as to words used in the instruments [21]. A pilot study was conducted to ensure the validity of the study instrument, and based on the findings from the pilot study, no changes were made to the questionnaire. The pilot study included 50 of the original sample of participants meeting the inclusion criteria who were recruited by the same methods used for the main sample size. The pilot study helped determine the time to complete the questionnaire and any difficulties during the application of the questionnaire in a different culture such as Saudi Arabia. All participants for the pilot study were required to have satisfactory English skills, as the questionnaires for the main study were in English, which is a common second language for both local and expatriate nurses.

Ethical approval was granted prior to commencement of the study from the RMIT University Human Research Ethics Committee (BSEHAPP 38-14) in Melbourne, Australia, the Institutional Review Board of the included hospitals under the Ministry of Health (FWA00018774) and the office of Research Affairs at King Faisal Research Centre (2152107) in Saudi Arabia. Data analysis was performed using the IBM Statistical Package for the Social Sciences version 22 (SPSS Inc., Chicago, IL, USA). The demographic data were analysed using frequency distribution. For the facilitator scale, the individual items were analysed by summing the average of ‘moderate to great extent’ and then ranking them. The item with the combined most responses for great or moderate facilitators was ranked first, and the item with the least responses was ranked last. For testing of the significant differences amongst each of the categories, a robust (Welch) one-way ANOVA was used. Multiple regression analysis was used where appropriate to assess which demographic variables had the largest impact on the facilitator score. Chi-square tests were used to analyse the demographic profiles of the participants. Independent sample *t*-tests were used to compare the mean values between groups of participants. The level of statistical significance was set at *p* ≤ 0.05.

## 3. Results

There were 1824 questionnaires that were retrieved from a total of 2650 eligible nurses, with a response rate of 69%. Most of the participants were female (82.7%), aged between 20 to 40 years (70.4%), expatriate (86.7%) and from the Philippines (47%), had between 6 and 10 years’ experience (29%), were clinical nurses (82.4%), had a bachelor’s degree (82.7%) and obtained their nursing qualification in the Asian region (50.1%) (Table 1).

The mean total facilitator score for all participants was 26.1 out of a total score of 32. Most participants responded with ‘to a moderate extent’ or ‘to a great extent’ for each of the eight facilitator items. Participants perceived all these items as strong facilitators of research utilisation, as responses varied by a maximum of 5% between the top-ranked and the bottom-ranked facilitators. Furthermore, participants who selected ‘no opinion’ compared with the ‘moderate to great extent’ category was low (Table 2). For the least-ranked facilitators of research utilisation, the most frequent were ‘increasing the time available for reviewing and implementing research findings’ (*n* = 338, 18.5%), ‘improving availability and accessibility of research reports’ (*n* = 318, 17.4%), ‘improving the level of understanding of research reports’ (*n* = 297, 16.3%) and ‘employing nurses with research skills to serve as role models’ (*n* = 283, 15.5%).

### Demographics

Across various demographics, the mean total facilitator score was compared to assess whether there were any differences. Males had higher facilitator scores (*M* = 26.9, *SD* = 4.4) compared to females (*M* = 25.9, *SD* = 5.8). For all participants, the highest-ranked facilitator of research utilisation was ‘advanced education to increase your research knowledge base’, whilst the most frequent least-ranked facilitator was ‘increasing the time available for reviewing and implementing research findings’. Participants aged 41–50 years had the highest mean total facilitator score compared with other age categories (Table 3). The differences in the mean total facilitator score were significantly different across the various age categories (*F* (3,1820) = 7.3, *p* < 0.001). Moreover, participants aged 20–30 years had a significantly lower facilitator score than those aged 31–40 years (*p* = 0.016; 95% CI: −1.7, −0.12) and 41–50 years (*p <* 0.001; 95% CI: −2.4, −0.7).

According to the type of nurses’ qualifications, there were statistically significant differences in the mean total facilitator score (*F* (2,1807) = 22.34, *p* < 0.001). In particular, the mean facilitator score increased with increasing education (Table 4), as participants with a master’s qualification had significantly higher facilitator scores compared with participants with a bachelor’s (*p* < 0.001; CI: −4.8, −1.01) and diploma qualifications (*p* < 0.001; 95% CI: −7.033, −3.02). Moreover, nurses with a bachelor’s qualification had a significantly higher facilitator score when compared with those with a diploma (*p* < 0.001; 95% CI: −3.03, −1.3).

Based on the region where the nurses’ attained their qualifications, there were statistically significant differences in the mean total facilitator score (*F* (3861.5) = 8.9, *p* < 0.001). Participants with qualifications from a Western region had higher mean total facilitator scores than participants with qualifications from the Middle East, India and Pakistan and Asian regions (Table 4). The facilitator score was significantly higher between participants who qualified in a Western region and the Middle East (*p* < 0.001; 95% CI: −4, −0.99) compared with India and Pakistan (*p* < 0.001, 95% CI: −3.9, −0.95). The facilitator score was also significantly different between participants who qualified in the Middle East and Asia (*p* = 0.008; 95% CI: −1.97, −0.2) and Asia, India and Pakistan (*p* = 0.008; 95% CI: 0.18, 1.85).

There were statistically significant differences in the mean total facilitator score amongst nurses with various experience levels (*F* (4,1391) = 8.9, *p* < 0.001), with increasing nursing experience resulting in a higher mean total facilitator score (Table 5). Comparison amongst the levels of experience demonstrated significant differences in facilitator scores between participants with 2 to 5 years and 11 to 15 years (*p =* 0.011; 95% CI: −2.4, −1.83) and those with greater than 20 years of experience (*p <* 0.001; 95% CI: −2.8, −0.54), and between participants with 6 to 10 years and 11 to 15 years (*p* = 0.001; 95% CI: −2.4, −0.4), 16 to 20 years (*p =* 0.039; 95% CI: −2.92, −0.04) and those with 20 or more years of experience (*p* < 0.001; 95% CI: −2.9, −0.8).

The mean total facilitator scores were compared across the various clinical roles of nurses. Nurse educators had the highest mean total facilitator score, followed by nurse managers and clinical nurses (Table 5). There were statistically significant differences in the mean total facilitator score amongst the different clinical roles occupied by nurses (*F* (2365.1) = 14.4, *p* < 0.001). These differences were statistically significant between nurse educators and nurse managers (*p* = 0.016; 95% CI: 0.27, 3.33) and clinical nurses (*p <* 0.001; 95% CI: 1.6, 4.1) and nurse managers and clinical nurses (*p* = 0.041; 95% CI: −2.04, −0.033).

## 4. Discussion

The demographic findings for age, gender, qualification and nursing experience are comparable to statistical data from other studies undertaken in Saudi Arabia [19,22,23]. Most of the participating nurses were expatriate; however, the frequency of expatriate nurses in this study at 87% is lower than reported by Omer [17] at 95%, higher than reported by Alqahtani and Jones [23] at 75% and higher than the Ministry of Health (2015) at 62%. These findings do demonstrate evidence of the Saudi Arabian government’s success in encouraging more Saudi nationals to undertake nursing education [24]. The finding of the largest group of nurses from the Philippines is not surprising, as they are the largest exporter of nurses worldwide [25]. This is largely due to a Philippines government-approved program of education of nurses for export [26], plus the poor working conditions in the Philippines that encourage nurses to seek work in other countries [25]. Moreover, recruitment agencies for nurses to work in Middle East countries are based in India and the Philippines [22,24,27]. It was not surprising that most nurses were clinical or bedside nurses followed by nurse managers and nurse educators, as this is comparable to any hospital nursing employment structure.

There are very few studies that have used a facilitator score at all or compared this score with demographic data. This study, however, identified significant relationships between facilitator scores and various demographic characteristics. Of note, the highest facilitator scores were achieved with nurses with a master qualification from the Western region, with nursing experience of greater than 20 years and being a nurse educator. Having a higher facilitation score indicates the identification of more facilitators to research utilisation. Master’s educated nurses are more aware of evidence-based practice and the need to utilise research, so it is not surprising that they had significantly higher facilitation scores compared with lower qualifications. Similarly, nurses with a master’s or doctoral degree were found to be strong predictors of higher EBP competency [28]. This is supported by Squires et al. [29], who found a positive association between nurses with a graduate degree and research utilisation.

The significantly higher facilitation scores for nurses from Western countries reflect the different emphasis in the curricula in these countries. There is more likely to be research content included in the curricula of Western countries, whilst the emphasis tends to be more on the English language, religion and culture for other countries [30]. In Saudi Arabia, however, a foundational research course is included in the more recent nursing curriculum, which introduces research, enabling nurses to conduct and apply research in their daily work practice. The impact of this change in the curriculum on nurses’ perception of facilitators of research utilisation was not explored due to insufficient numbers of Saudi Arabian nurses included in the study.

The finding of nurses with more experience having significantly higher facilitator scores could possibly be related to these nurses being able to identify more facilitators to research utilisation purely based on their nursing experience. This is supported by Kang [31], who found that in nurses with less experience, the barriers to research utilisation were higher. Correspondingly, Oh [32] found barriers to research utilisation were significantly related to professional status and length of clinical experience. Oh [32] also found that staff nurses with less than 10 years of clinical experience perceived greater barriers to research utilisation.

In terms of the various clinical roles of nurses, nurse educators had significantly higher facilitator scores. These findings may be explained by the role of the nurse educator, which is to encourage the application of evidence-based practice and to undertake research or participate in the process. Additionally, nurse educators are more likely to have undertaken further education for their role and, therefore, are expected to have knowledge of research. This is supported by other studies that found that clinical nurses had higher perceived barriers to research utilisation than nurse managers or nurse educators [16,32]. These findings are supported by a focus on clinical and specialised roles and a lack of public health education for nurses in Saudi Arabia. This has prompted calls for inclusion in the nursing curriculum of a public health syllabus that facilitates EBP and research utilisation [33].

The factors that were identified as strong facilitators of research by more than 80% of the nurses in each of the five hospitals included advanced education, providing colleague support, more clinically focused research and employing nurses with research skills. For the least likely facilitators, these included time for reviewing and facilitating research, availability of research, improving the level of understanding of research reports, enhancing managerial support for research implementation and employing nurses with research skills to serve as role models.

The results of this study confirm the importance and value of education for the utilisation of research by nurses. Similarly, Sanjari et al. [34], in a systematic review of barriers and facilitators of nursing research utilisation in Iran, found education was a major facilitator. In an integrative international review of 42 studies of barriers and facilitators of research utilisation by nurses, the most common facilitator identified was increased nurse education and research utilisation awareness [35]. Likewise, Cline et al. [8] identified that for nurses working in a tertiary care children’s hospital, education in research was an important facilitator for research utilisation as well as research mentors. For nurses from a range of hospital and community settings in The Bahamas, the most frequently identified facilitator for the implementation of evidence-based practice was education in research methods followed by knowledge about evidence-based practice [36]. This contrasts with the findings of Omer [19], who used open-ended questions to ascertain facilitators of research utilisation. Omer [19] found that an increase in administrative support was important for nurses for research availability and implementation, which contrasts with this study that identified these factors as least facilitative to research utilisation. A factor that could help explain the finding for this study of administrative support as a lower-ranked facilitator was the establishment of a research culture for nurses in hospitals. Several participants in this study recognised a research culture in their respective hospital and reported participating in education sessions on research as part of in-service education and receiving financial assistance to attend conferences. However, in other studies, managerial support has been identified as the most important facilitator [12,14]. In a study to identify barriers and facilitators to research use amongst Jordanian nurses working in critical care units, only 26% of the participants reported facilitators, and of these, organisational support was the most reported [37].

Similar to the findings for this study, Srijana et al. [38] noted that conducting research was an important facilitator. Srijana et al. [38] found that the initiation of nursing research projects, education on research methods and the provision of funding for the conduct of research were top facilitators for the utilisation of research.

Interestingly, increasing time for reviewing and implementing research was ranked as the least facilitative item from this study but was perceived by nurses as the most important facilitator for several other studies [18,20,39]. The result of the provision of colleague support as the main facilitator for this study is also supported by the findings of several other studies [11,17,20].

For this study, the data suggest that nurses in Saudi Arabia recognise the value of advanced education and research as facilitators of research utilisation. The findings of this study indicate that the more educated the nurse, the more aware they are of the need for research utilisation. It is also evident from the literature of the value of advanced education, as nurses with higher degrees can create a culture of evidence-based thinking that can be empowered by education programs prepared and provided by these nurses, as well as increasing the number of comparable academically prepared nurses [40]. Indeed, it has been demonstrated that if nurses with doctoral degrees collaborate with organisation leaders to conduct clinically related research or use published research to resolve problems in clinical settings, this can influence nursing practice to be more evidence-based [5]. These findings and those of this study suggest that nurses with higher degrees should be allocated to roles that allow them to make changes to nursing practice through the application of research findings.

A strength of this study was the sample size (1824) and that this accounted for a large number (69%) of the total eligible nurses (2650) from the five hospitals included in the study. This is especially the case when comparing this study to similar research undertaken in Saudi Arabia by Omer [19], which had a smaller sample size (413), lower response rate (34%) and only one hospital site. The large dataset also provided an opportunity to undertake numerous correlations that have not been undertaken to this extent before. In particular, the investigation of the demographic effects on facilitators of research utilisation.

A limitation of the study included the use of a self-reported questionnaire. With the utilisation of these types of questionnaires, it is difficult to avoid response biases because of poor understanding of the questionnaire items or participants answering questions based on perceived socially desirable responses. Nevertheless, it should be noted that for the facilitator questionnaire items, the frequency of ‘no opinion’ results compared with the findings for the ‘moderate to great extent’ was low. The use of a convenience sample is another limitation, as the results may not be representative for all nurses in Saudi Arabia, although, as previously discussed, the study sample size was large. Another limitation was the inclusion of only hospitals located in Riyadh, which may not be representative of other areas of Saudi Arabia.

As acknowledged in the literature, a definitive solution of how best to facilitate research utilisation has yet to be found [41]. However, this study offers an evidence base for nursing education in Saudi Arabia for the identification of facilitators to enhance research utilisation. These facilitators include strategies to assist in the conduct and application of research in nursing practice as well as improving knowledge of research and reading skills to facilitate interpretation of research. This can be achieved through leadership and organisational change, which is supportive of strengthening research education in the nursing curriculum as well as in the hospital setting.

## 5. Conclusions

There is a paucity of literature analysing facilitators of research utilisation among nurses, and this study extends much of that knowledge. The facilitators of research utilisation among nurses identified in this study are similar to those of previous studies in other countries. Nurses’ perceptions of factors facilitating the utilisation of research in practice relate mainly to education, including employing nurses with higher education and research skills, as well conducting clinically focused research and colleague support. In terms of demographic characteristics, nurses with a qualification from a Western region with a master’s qualification and who were nurse educators, older and had more experience had the highest facilitator scores. Further studies are needed to further explore nurses’ perceptions of the facilitators of research utilisation and to develop formal strategies for implementation. Recommendations for this study include a strengthening of research education in nursing education programs as well as through continuing professional education. This manuscript complies with STROBE.

## Figures and Tables

**Table 1:** Demographics of the respondents (*n* = 1824).

Characteristics	Frequency (*n*)	Percentage (%)
**Age (years)**		
20–30	616	33.8
31–40	669	36.7
41–50	413	22.6
51–64	126	6.7
**Gender**		
Male	315	17.3
Female	1509	82.7
**Country of birth**		
KSA	242	13.3
Philippines	857	47.0
India	501	27.5
South Africa	50	2.7
Jordan	62	3.4
Pakistan	35	1.9
Egypt	4	0.2
Australia	7	0.4
USA	13	0.7
Canada	8	0.4
Malaysia	40	2.2
Lebanon	2	0.1
UK	3	0.2
**Experience (years)**		
2–5	369	20.2
6–10	544	29.8
11–15	418	22.9
16–20	175	9.6
>20	318	17.4
**Current role**		
Clinical	1503	82.4
Manager	231	12.7
Educator	89	4.9
**Qualification**		
Hospital certificate	14	0.8
Diploma	269	14.7
Bachelor	1508	82.7
Master	33	1.8
**Qualification obtained by region**		
Middle East	292	16.0
Asia: Philippines and Malaysia	913	50.1
India/Pakistan	535	29.3
Western: America, Europe, South Africa and Australia	84	4.6

**Table 2:** Ranking of facilitators of research utilisation (moderate to great extent).

Facilitator of Research Utilisation Questionnaire Items	Moderate–Great Extent (*n*)	Percentage	Item Mean	Item SD	No Opinion *n* (%)
Advanced education to increase your research knowledge base	1542	84.6	3.32	0.87	26 (1.4)
Providing colleague support network/mechanisms	1526	83.7	3.29	0.88	28 (1.5)
Conducting more clinically focused and relevant research	1518	83.2	3.28	0.88	32 (1.8)
Employing nurses with research skills to serve as role models	1498	82.1	3.30	0.94	43 (2.4)
Enhancing managerial support and encouragement of research implementation	1493	81.9	3.22	0.96	60 (3.3)
Improving the understandability of research reports	1486	81.5	3.24	0.92	41 (2.2)
Improving availability and accessibility of research reports	1463	80.2	3.22	0.94	43 (2.4)
Increasing the time available for reviewing and implementing research findings	1448	79.4	3.17	0.94	38 (2.1)

**Table 3:** Mean total facilitator score for nurses’ age categories.

Age (Years)	*n*	Mean	SD
20–30	616	25.30	5.7
31–40	669	26.20	5.7
41–50	413	26.80	4.9
51–64	126	26.50	6.2

**Table 4:** Mean total facilitator score for nurses’ region where qualification or qualifications attained.

Characteristics	*n*	Mean	SD
**Qualification**			
Diploma	269	24.20	2.32
Bachelor	1508	26.30	3.88
Masters	33	29.20	4.02
**Region**			
Middle East	292	25.40	5.12
Asia: Philippines and Malaysia	913	26.50	6.04
India and Pakistan	535	25.40	6.93
Western	84	27.90	4.58

**Table 5:** Mean total facilitator score for nurses’ experience levels and clinical roles.

Characteristics	*n*	Mean	SD
**Years of experience**			
2–5	369	25.40	5.76
6–10	544	25.20	6.99
11–15	418	26.70	3.13
16–20	175	26.70	4.81
>20	318	27.10	5.34
**Role**			
Clinical nurse	1504	25.70	5.90
Nurse manager	231	26.80	5.92
Nurse educator	89	28.60	4.71

## Data Availability

Data supporting the results from this paper are stored at the RMIT University research repository.
